# An *in vitro* and *in vivo* study of plasma treatment effects on oral biofilms

**DOI:** 10.1080/20002297.2019.1603524

**Published:** 2019-04-19

**Authors:** Qing Hong, Xiaoqing Dong, Meng Chen, Hongmin Sun, Liang Hong, Yong Wang, Hao Li, Qingsong Yu

**Affiliations:** a Center for Surface Science and Plasma Technology, Department of Mechanical and Aerospace Engineering, University of Missouri, Columbia, MO, USA; b Nanova, Inc., Columbia, MO, USA; c Department of Internal Medicine, University of Missouri, Columbia, MO, USA; d Department of Pediatric and Community Dentistry, College of Dentistry, University of Tennessee Health Science Center, Memphis, TN, USA; e Center for Research on Interfacial Structure & Properties, School of Dentistry, University of Missouri-Kansas City, Kansas City, MO, USA

**Keywords:** Oral biofilm, *streptococcus mutans*, rat model, non-thermal atmospheric gas plasmas, biofilm recovery, metabolic activity, host defense

## Abstract

Management of dental plaque/biofilms is critical to maintain oral health. The objective of this study is to investigate the treatment effects of non-thermal atmospheric gas plasmas on oral biofilm formation and recovery under *in vitro* and *in vivo* conditions. *Streptococcus mutans* biofilms, a significant contributor to tooth decay, were cultured and treated by plasma. It was found that plasma treatment not only significantly reduced the *in vitro* biofilms, but also increased the metabolic activity of the bacteria in the biofilms. As compared with untreated control group, the cell metabolic activity, as measured by MTT assay, increased by 273%, and the aconitase activity increased by 446% for the plasma-treated group. The increased metabolic activity of the plasma-treated biofilm bacteria enhanced their susceptibility to antibiotic and host defense. An *in vivo* animal model using a total of 60 female rats (19 days old) were used to evaluate the anti-caries effects on the molars by 2 min of plasma treatment. It was found that, 6 months after the plasma treatment, the decayed surfaces were reduced by 62.5% on the upper molars and by 31.6% on the lower molars as compared with the untreated upper and lower molars, respectively. These *in vitro* and *in vivo* data demonstrate that the physiological state change of the biofilm due to plasma treatment provided benefit to caries control and prevention.

## Introduction

Dental plaque is the collection of microorganisms adhering to a tooth surface as a biofilm. Various species of bacteria are held together by sugary molecular strands, which is termed extracellular polymeric substances (or EPS). EPS allow biofilms to develop complex three-dimensional, resilient, and attached communities. As a widely studied biofilm, dental plaque displays all of the characteristic features of a typical biofilm, such as open architecture [1], enhanced tolerance to antimicrobials [2], and enhanced virulence [3]. Numerous studies have shown that dental plaque is one of the significant factors causing dental caries, periodontitis, and other oral infections [4].

Due to its harmfulness, management of dental plaque is critical to maintain oral health. The current therapeutic strategy to control dental plaque usually involves mechanical removal and the use of chemical agents. Scaling (removal of calculus and plaque), root planning (removal of necrotic tooth tissue on the root surface), and surgery (to remove tissue and reduce pocket depth) are the most common removal methods of supra and subgingival plaque in periodontal therapies [5]. The mechanical procedures undoubtedly remove most organisms colonizing the tooth surface. However, given the rapid multiplication rate of bacteria, it is not surprising that the majority of bacteria return to almost baseline levels shortly after the removing procedures. Data in the literature suggest that the return to baseline total counts might occur within 4–8 days [6,7].

Besides the traditional mechanical means, topical and systemic antimicrobial agents can be used in mouthwashes, toothpastes, pills or other delivery systems. The chemotherapeutic agents could reduce plaque through (1) affecting initial colonization, (2) inhibiting plaque development and metabolism, or (3) by reducing existing development [8]. A wide range of antimicrobial agents, such as chlorhexidine [9], triclosan [10], and ciprofloxacin [11], have been formulated into oral care products. However, the resistance of biofilm to antimicrobial agents is a significant concern [12].

Non-thermal atmospheric plasma has been considered as an alternative method to overcome the limitation of conventional biofilm removal methods. Due to its enhanced plasma chemistry, non-thermal plasma contains many active plasma species including various charged particles and reactive oxygen species (ROS) that are effective in inactivating bacteria [13]. Non-thermal plasma technology has demonstrated a promising capability in disinfecting dental plaque [14]. Non-thermal atmospheric plasma is very effective against Gram-positive bacteria [15], Gram-negative bacteria [16], and fungi [17]. It has also been confirmed that non-thermal plasma does not cause any pathological changes in the normal mucosa [18]. Application of plasma treatment was much more effective than chlorhexidine digluconate (CHX) in disinfecting biofilms [19]. It was found that plasma could penetrate into biofilms and effectively deactivate all the bacteria in 15 μm thick biofilms [16]. Pei et al. reported that plasma species from an air plasma jet could even penetrate to the bottom layer of a 25.5 μm-thick *Enterococcus faecalis* biofilm and produce strong bactericidal effects [20]. Based on its *in vitro* antibacterial activity, non-thermal atmospheric plasma could also be an effective approach against bacterial biofilms in root canal systems [21]. Very recently, Koban et al. [22] studied the synergistic effect of plasma treatment and different agents in dentistry on killing multispecies oral biofilms, which reflect the natural environment of many pathogens in clinical settings.

It should be pointed out that most of the research has focused on the instant plasma disinfection against biofilm, but not the biofilm behavior after plasma treatment. It is difficult to completely eradicate biofilm by conventional dental plaque removal methods because the bacterial microcosms recover rapidly, limiting the efficacy of conventional therapies. Pratten [23] reported that pulsing chlorhexidine treatment initially achieved substantial killing of biofilm bacteria, but the viability of the biofilms subsequently increased. In this study, non-thermal atmospheric plasma treatment of *Streptococcus mutans* biofilms was found to have the capability to limit biofilm recovery and caries development.

## Materials and methods

### Biofilm preparation and plasma treatment

One colony of *S. mutans* (ATCC 700610, ATCC, Manassas, VA) on an agar plate was selected and cultured in 5 ml Tryptic Soy Broth (TSB, BD 211825, Franklin Lakes, NJ) medium overnight. The cultured *S. mutans* suspension was then diluted 1:200 with TSB medium (containing 0.5% sucrose) [24]. Sterilized stainless steel wafers (1 × 1 cm) were placed in a 24-well plate with 1 ml of the diluted *S. mutans* suspension added into each well. The plate was then incubated at 37°C for 8 h. The wafers with biofilms on the surface were rinsed with phosphate-buffered saline (PBS) three times in order to remove non-adherent bacteria. The biofilm was subsequently treated by atmospheric plasma, subsequently transferred to a new 24-well plate, and re-cultured with 1 ml sucrose-containing TSB medium at 37°C for 16 h. Another group of biofilms without plasma treatment was used as a control.

A low-temperature atmospheric plasma brush consists of a tube with a converging nozzle at one end. In the nozzle, two tungsten wires serve as a ground electrode and a cathode that is connected with a ballasted resistor. Three thousand standard cubic centimeters per min (sccm) of argon (99.9% minimum purity) and 30 sccm of ultra-high purity oxygen were premixed through the tube and delivered to the nozzle. An electric field was applied across the electrodes by a DC power supply (Spellman SL60, Hauppauge, NY) and plasma was created. The power was kept around 3.0 W, i.e. 6.0 mA and approximately 0.50 kV. Detailed information on the plasma device has been reported in our previous study [25].

### Biofilm assays

For biofilm formation, the prepared biofilms were washed with PBS three times and stained with 500 ul 2.3% crystal violet (CV) (Sigma-Aldrich, St Louis, MO) for 20 min. The stained biofilms were rinsed with PBS five times. The wafers were then transferred to a new plate and 1 ml ethanol was added to each well to dissolve the CV. The concentration of CV was determined by measuring OD_595nm_ of 1:10 diluted samples with a microplate reader (SPECTROstar Nano, BMG Labtech, Cary, NC). The percentage of biofilm formation was calculated against the mean of untreated biofilm control samples.

For biofilm slime measurements [26], the prepared biofilms were washed with PBS three times and fixed with Carnoy’s solution (Fisher Scientific, Fair Lawn, NJ) for 30 min and stained with 0.1% toluidine solution (Sigma-Aldrich) for 30 min. The samples were subsequently transferred to a new 24-well plate and incubated in 0.2 M NaOH solution for 1 h in an 85°C water bath. The optical density was measured at a wavelength of 590 nm.

For CFU counting by the plate counting method [26], the biofilms on wafers were washed with PBS three times and transferred to 5 ml tubes with 2 ml PBS. The biofilms were ultra-sonicated and vortexed for 20 s for five cycles to detach the bacteria from the wafers. The bacterial cells in PBS were diluted and quantified using the spread plate technique.

For bacterial viability assessment, the biofilms were first washed with PBS three times and then stained with 400 µl MTT (define) solution for 3 h. The wafers were then transferred to a new plate. A mixture (400 µl) of DMSO and ethanol (1:1) was added into each well. The plate was placed on a shaker for 30 min to completely dissolve the crystal, which concentration was later measured by OD_570nm_. The percentage of biofilm viability was calculated against the mean of the untreated control biofilms. Moreover, the MTT activity per cell was normalized by dividing the OD value with the CFU count.

### Quantitative PCR (qPCR) analysis

For bacteria quantitation by qPCR [27], the biofilms prepared on wafers as described in section 2.1 were washed three times with PBS and put into 5 ml tubes with 2 ml PBS. The bacteria were detached from the wafers with ultrasonic bath treatment and then digested with lysostaphin and proteinase K. An EZ cell DNA isolation kit (EZ Bioresearch, St. Louis, MO) was used to isolate genomic DNA. SYBR green-based quantitative PCR (qPCR) was performed with DNA samples. The amount of DNA was quantitated by the *16S rRNA* gene. The primers for the *16S rRNA* gene were GTAGCGGTGAAATGCGTAGATA (forward) and CGCTAGAGTGCCCAACTTAAT (reverse). The relative DNA concentrations were calculated using the 2^−^∆∆CT method (give ref).

### Aconitase activity assay

One ml of the detached bacteria suspension from the prepared biofilms was transferred to a 1.5 ml centrifuge tube and centrifuged for 5 min at 14,000 rpm. The pellet was collected to assess the aconitase activity using the aconitase activity colorimetric assay kit (Biovision Inc, Milpitas, CA). In this assay, the aconitase activity was measured by the amount of isocitrate, which is converted from citrate by the catalysis of aconitase. The aconitase activity was normalized by dividing it by the CFU amount.

### Oxidative stress tolerance of plasma-treated biofilms

Pre-cultured biofilms were treated by atmospheric plasma for 1 min and then transferred to a new 24-well plate for re-culturing at 37°C for 16 h with 1 ml TSB medium containing different oxidative chemicals. As an untreated control, 8 h old biofilms were used and cultured at the same condition as the plasma-treated group. Hydrogen peroxide (Sigma-Aldrich) and paraquat (Sigma-Aldrich) were utilized as oxidative stress producers. The final hydrogen peroxide concentration was 0.004% and the final parquet concentration 200 mM in the culture medium. The biofilms were then evaluated using the CFU assay.

### Antibiotic susceptibility of plasma-treated biofilms

Biofilms (8 h old) with and without plasma treatment were incubated in TSB medium (containing 5% sucrose) with 0 or 8 µg/ml ciprofloxacin (Sigma-Aldrich) at 37°C for 16 h. CFU assay was then used to evaluate the biofilms.

### *In vivo* anti-caries effect on the experimental rat model

To study the *in vivo* anti-caries effects of the plasma treatment, a total of 60 Sprague–Dawley female rats (19 days old) purchased from the Charles River Breeding Laboratory were used. The rat model study was conducted by following Animal Research Protocol number 1970 approved by the Institutional Animal Care & Use Committee, Health Science Center at the University of Tennessee. All the rats were fed with a sucrose-containing diet (D12450B; Research Diet) throughout the entire experiment. On day 1, all the rats were anesthetized with ketamine/xylazine 80–100/10mg/kg, IP. The left side molars (including both maxillary and mandibular molars) were treated by uniformly scanning the plasma flame on the tooth surface (including outer, inner, chewing, and adjacent surfaces) for 2 min for each molar. The right side molars received no plasma treatment and served as control. After 6 months, all the rats were euthanized for further investigations. Both jaws of the rats were dissected and fixed in 10% neutralized formalin for caries scoring. All molars of the rats were examined under a dissecting microscope, and carious lesions were scored according to the Keyes method [28].

## Results

### Effects on biofilm recovery

**Figure 1.**
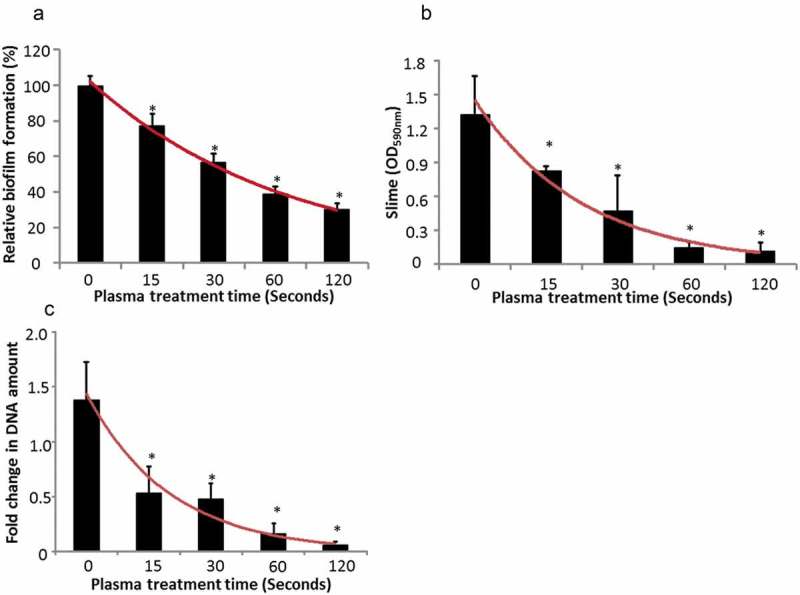
Biofilm recovery after plasma treatments with different treatment time periods. (**a**) Crystal violet staining of biofilm formation, (**b**) slime production of biofilms, (**c**) qPCR analysis of biofilm.

Higher biofilm inhibition was achieved by increasing the plasma treatment time (Figure 1). Biofilm was reduced by 70% on stainless steel wafers after 2 min of plasma treatment characterized by CV staining measurement (Figure 1(a)). The slime production of biofilm decreased from OD = 1.32 to OD = 0.12 as the plasma treatment time increased from 0 min to 2 min (Figure 1(b)). Moreover, the same trend was observed in DNA quantity. The biofilm receiving 2 min of plasma treatment preserved 6% of DNA of that of untreated biofilm after recovery (Figure 1(c)).

### Effects on metabolism activity

**Figure 2.**
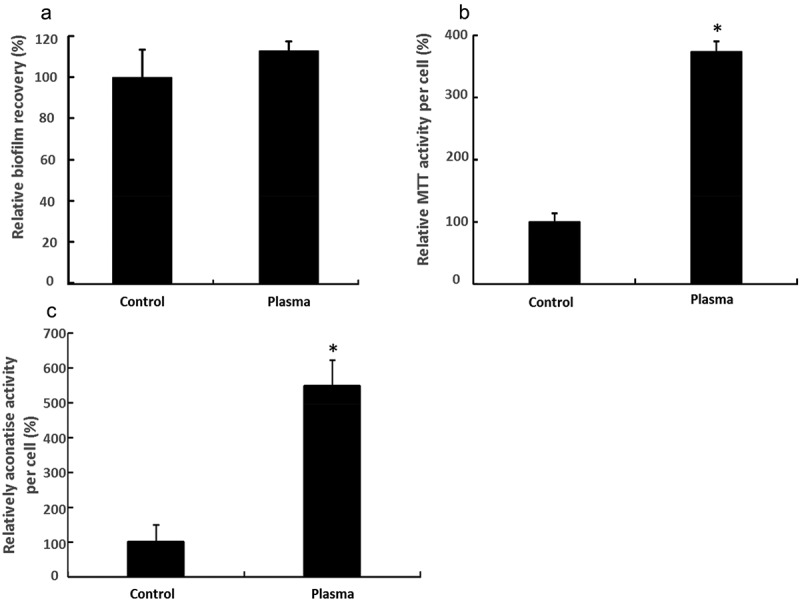
Metabolic activity of biofilms. (**a**) Total MTT activity of recovery biofilms, (**b**) relative MTT activity per cell, (**c**) relative aconitase activity per cell.

Bacteria in biofilms after plasma treatment demonstrated a 13% increase of the MTT value in comparison with the control group (as shown in Figure 2(a)). The result seems to be in contradiction with biofilm measurement by CV staining, biofilm slime assay, and genomic DNA quantitation (Figure 1). When the MTT results were presented in the form of relative viability per cell, as seen in Figure 2(b), plasma-treated bacteria illustrated 273% higher viability than non-treated bacteria. This result suggested that bacteria in plasma-treated biofilms may be more metabolic active than bacteria in untreated biofilm because MTT affects cell metabolic activity. The aconitase activity was measured to test this hypothesis. As shown in Figure 2(c), the aconitase enzyme activity of bacteria in plasma-treated biofilm increased by 446% as compared with the control group.

### Effects on host defense and antibiotic treatment

**Figure 3.**
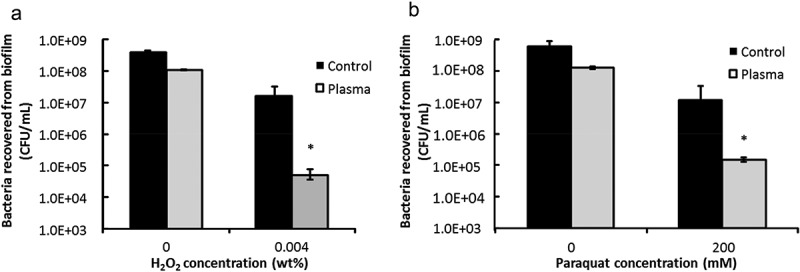
Biofilm responses to oxidative stresses. (**a**) CFU counting for biofilm amount under the oxidative stress of hydrogen peroxide, (**b**) CFU counting for biofilm amount under the oxidative stress of paraquat.

H_2_O_2_ was used as an oxidative stress inducer to test the response of plasma-treated biofilms to oxidative stress, which is an important part of host innate immunity [29]. Figure 3(a) displays the comparison of biofilm recovery with H_2_O_2_ stress between the plasma group and the control group. Under 0.004% H_2_O_2_ treatment, four log unit of reduction of recovered biofilm was observed for plasma-treated biofilms, while merely 1.8 log unit of reduction of recovered biofilm was observed for non-treated biofilm at the same H_2_O_2_ concentration, as measured by CFU assay. Paraquat was used as another oxidative stress inducer. The difference in oxidative stress response between non-treated and plasma-treated biofilms was shown in Figure 3(b). Plasma-treated biofilm was also less resistant to paraquat treatment. There was a 2.7 log reduction of bacteria (CFU) in plasma-treated biofilms while only a 1.5 log reduction in non-treated biofilm was observed at 200 mM paraquat treatment (Figure 2(b)).

**Figure 4.**
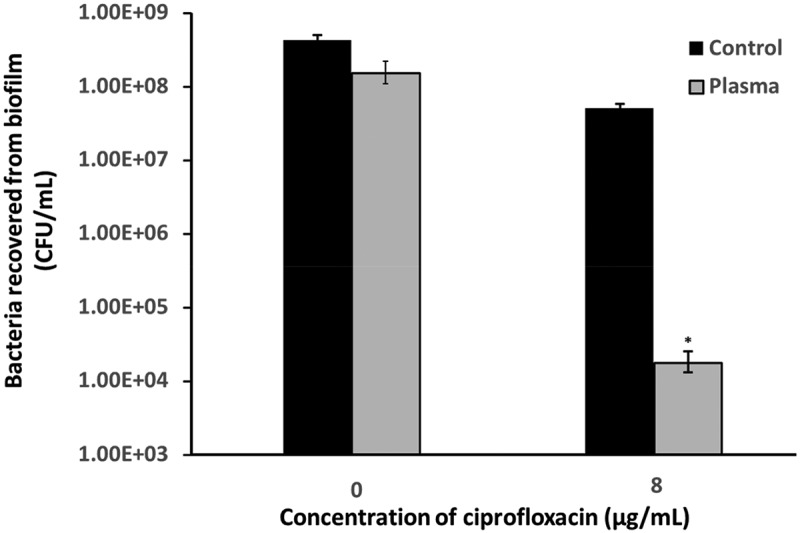
Biofilm response to ciprofloxacin treatment measured by CFU counting.

Biofilm response to antibiotics was studied by treating the bacteria in biofilm with 8 µg/ml ciprofloxacin. The difference of biofilm response between the control and plasma group are presented in Figure 4. When plasma and antibiotic were combined, there was a 4.4 log reduction by CFU counting as compared to that of the control group. In contrast, there was only 0.9 log CFU reduction in the group without plasma treatment as compared to that of the control group.

### Effects on *in vivo* caries development

**Figure 5.**
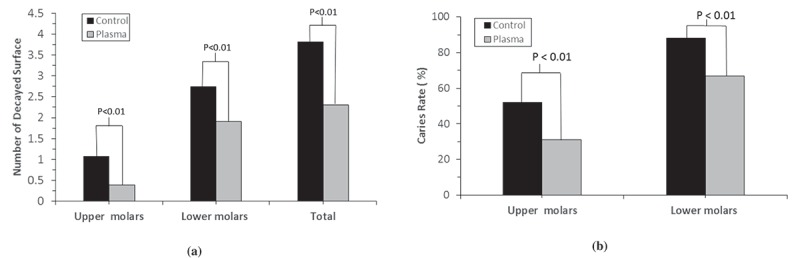
(**a**) Number of the decayed surfaces on upper and lower molars; (**b**) Caries rate on upper and lower molars.

Figure 5(a) summarizes the numbers of decayed tooth surfaces of the rats. Plasma-treated sides demonstrated less decay than the control sides in both upper and lower molars. The decayed surfaces on the upper molars with plasma treatment were reduced by 62.5%, compared with those of the untreated upper molars. A similar trend was observed on the lower molars, where there was a reduction of 31.6% after plasma treatment. Moreover, caries was also less prevalent on plasma-treated sides than on control sides, as shown in Figure 5(b). The caries rate was 31% for plasma treated upper molars as compared to the 52% for untreated controls. Similar trends was observed for lower molars with 67% caries rate for plasma treated ones compared with 88% for untreated controls. The difference was statistically significantly.

## Discussion

Many studies have demonstrated the plasma disinfection capability against bacteria and biofilms [15,16,25]. However, most of the studies focused on the instant efficiency of plasma disinfection. Few studies investigated the status of the recovered bacteria after plasma treatment. In this study, the biofilm formation and recovery after plasma treatment were investigated. The plasma-treated biofilms were re-cultured in the media for 16 h prior to biofilm assays. The results of CV staining, biofilm slime production and genomic DNA quantitation demonstrated that the amount of recovered bacteria in plasma-treated biofilms was significantly decreased. These findings suggest that plasma treatment not only inactivates biofilm instantly, but also inhibits the biofilm growth after the treatment. Since it is very difficult to completely prevent the biofilm from returning after removing treatments, the inhibition of biofilm recovery could be critical in effectively controlling biofilms.

MTT assay was applied to assess the viability of recovered biofilms. It was found that plasma-treated biofilm exhibited higher viability than untreated biofilm controls (Figure 2(a)), even though there were fewer cells observed in the plasma-treated biofilms than in the untreated biofilms (Figure 1). MTT is a widely used assay to estimate the cell number by assessing the cell metabolic activity [30]. The MTT assay results suggested that plasma treatment might increase the metabolic activity of bacteria in the biofilms.

In order to test this hypothesis, the activity of aconitase from the tricarboxylic acid (TCA) cycle in a crude lysate of *S. mutans* biofilm was studied. The function of aconitase is well known for catalyzing the conversion of citrate into isocitrate. This conversion plays a critical role in the TCA cycle, which is a central pathway of metabolism [31]. Therefore, aconitase activity is commonly used as a biomarker for metabolism [32]. In this study, it was found that the aconitase activity increased by 446% in the plasma-treated biofilm, suggesting higher bacterial metabolism in the plasma-treated biofilms than in the untreated controls. This result is in agreement with the results of the MTT assays.

Due to the reduced metabolic activity of bacteria in regular biofilms, it is difficult for the host to adequately attack and destroy infectious biofilm populations [33]. It provides passive protection against antibiotics and host defenses [34]. Given the higher metabolic activity of bacteria in the plasma-treated biofilms, we hypothesized that the plasma-treated biofilms might be more susceptible to antibiotics and host defenses. Ciprofloxacin is widely used in treating biofilm with antibiotics. The primary mechanism of action of ciprofloxacin is inhibition of the activity of A subunit of DNA gyrase, which leads to termination of chromosomal replication and to interference with cell division and gene expression [35]. The results of the CFU counting indicated that, after plasma treatment, the plasma-treated biofilms were less resistant to ciprofloxacin than the untreated biofilms.

Besides the antibiotic, host defense is also critical to prevent biofilm infection [36]. One of the important host defense mechanisms is the generation of reactive oxygen species (ROS) by polymorphonuclear leukocytes (PMNs) to mediate defense against bacterial pathogens [37]. The production of ROS, chemically reactive molecules containing oxygen, represents an essential arm of the innate immune system [38]. Phagocytic cells, especially macrophages and neutrophils, generate an extensive amount of ROS to kill bacteria [39].

Hydrogen peroxide (H_2_O_2_) and paraquat are two widely used sources to provide ROS in *in vitro* experiments. In this study, these two ROS sources were used to assess the response of plasma-treated biofilms to oxidative stress. The antimicrobial action of hydrogen peroxide is not due to its properties as a molecule, but primarily to the production of singlet oxygens, superoxide radicals, and the hydroxyl radical [40]. These ROS can cause irreversible damage to host cell components such as enzymes, membrane constituents and DNA [41]. Paraquat is often used to catalyze the formation of ROS, more specifically, the superoxide free radicals, which are biologically quite toxic and are deployed by the immune system to kill invading bacteria [42]. The superoxide free radicals could cause potentially toxic reactions such as peroxidation of polyunsaturated lipid, depolymerization of hyaluronic acid, inactivation of proteins and damage to DNA [43].

The plasma-treated biofilms displayed significantly lower tolerance to H_2_O_2_ and paraquat, compared with the untreated biofilms. Biofilms are more resistant to the attacking and killing by the host immune systems than planktonic bacteria, contributing to the increased virulence of biofilm infections [44]. Our results suggest that the plasma-treated biofilm could be well controlled by the host immune system due to the increase of metabolism of the biofilms induced by the plasma treatment. This was verified by the results in the *in vivo* rat model study. Both upper molars and lower molars treated with plasma presented much less decayed surface and lower caries rate than the untreated control group. These rat model data further demonstrate that the physiological state change of the biofilm due to plasma treatment provided the benefit to caries control and prevention. In clinical, 0% to 50% ADA (American Dental Association) CCS (Caries Classification System) initial pit-and-fissure caries lesions could exhibit histologic dentin penetration, and 50% to 88% ADA CCS moderate pit-and-fissure caries lesion may penetrate histologically to dentin [45] and thus cause permanent damages to tooth structure. Considering these probabilities, the much less decayed surfaces and lower caries rates observed on plasma-treated rat molars indicate that the non-thermal plasmas technology could be used as an innovative solution to treat and control oral diseases caused by biofilms.

## Conclusion

In conclusion, plasma treatment could increase the metabolic activity of oral biofilms, and make the biofilms more susceptible to antibiotic and oxidative stresses. As a result, the recovery of the plasma-treated biofilms could be more successfully controlled by antibiotics and the host immune system. This conclusion was further supported by the results obtained with a 6 month *in vivo* study using a rat model, in which much less decayed surfaces and lower caries rates were observed on plasma-treated rat molars than that on the untreated controls. In other words, the physiological state change of the biofilm due to plasma treatment provided the benefit to caries control and prevention.
